# HIV Antibody Profiles in HIV Controllers and Persons With Treatment-Induced Viral Suppression

**DOI:** 10.3389/fimmu.2021.740395

**Published:** 2021-08-26

**Authors:** Kai Kammers, Athena Chen, Daniel R. Monaco, Sarah E. Hudelson, Wendy Grant-McAuley, Richard D. Moore, Galit Alter, Steven G. Deeks, Charles S. Morrison, Leigh A. Eller, Joel N. Blankson, Oliver Laeyendecker, Ingo Ruczinski, Susan H. Eshleman, H. Benjamin Larman

**Affiliations:** ^1^Division of Biostatistics and Bioinformatics, Department of Oncology, Sidney Kimmel Comprehensive Cancer Center, Johns Hopkins University School of Medicine, Baltimore, MD, United States; ^2^Department of Biostatistics, Johns Hopkins University Bloomberg School of Public Health, Baltimore, MD, United States; ^3^Department of Pathology and the Institute for Cell Engineering, Johns Hopkins University School of Medicine, Baltimore, MD, United States; ^4^Department of Pathology, Johns Hopkins University School of Medicine, Baltimore, MD, United States; ^5^Department of Medicine, Johns Hopkins University School of Medicine, Baltimore, MD, United States; ^6^Department of Medicine, Ragon Institute of Massachusetts General Hospital (MGH), Massachusetts Institute of Technology (MIT) and Harvard, Cambridge, MA, United States; ^7^Department of Medicine, University of California, San Francisco (UCSF), San Francisco, CA, United States; ^8^Behavioral, Epidemiologic and Clinical Sciences, Family Health International (FHI) 360, Durham, NC, United States; ^9^U.S. Military HIV Research Program, Walter Reed Army Institute of Research, Silver Spring, MD, United States; ^10^Henry M. Jackson Foundation for the Advancement of Military Medicine, Bethesda, MD, United States; ^11^Laboratory of Immunoregulation, National Institute of Allergy and Infectious Diseases, National Institutes of Health, Baltimore, MD, United States

**Keywords:** HIV control, viral load set point, antibody profiling, phage display, VirScan

## Abstract

**Introduction:**

Low HIV viral load is associated with delayed disease progression and reduced HIV transmission. HIV controllers suppress viral load to low levels in the absence of antiretroviral treatment (ART). We used an antibody profiling system, VirScan, to compare antibody reactivity and specificity in HIV controllers, non-controllers with treatment-induced viral suppression, and viremic non-controllers.

**Methods:**

The VirScan library contains 3,384 phage-displayed peptides spanning the HIV proteome. Antibody reactivity to these peptides was measured in plasma from a Discovery Cohort that included 13 elite controllers, 27 viremic controllers, 12 viremic non-controllers, and 21 non-controllers who were virally suppressed on ART. Antibody reactivity to selected peptides was also assessed in an independent cohort of 29 elite controllers and 37 non-controllers who were virally suppressed on ART (Validation Cohort) and in a longitudinal cohort of non-controllers.

**Results:**

In the Discovery Cohort, 62 peptides were preferentially targeted in HIV controllers compared to non-controllers who were virally suppressed on ART. These specificities were not significantly different when comparing controllers *versus* viremic non-controllers. Aggregate reactivity to these peptides was also high in elite controllers from the independent Validation Cohort. The 62 peptides formed seven clusters of homologous epitopes in env, gag, integrase, and vpu. Reactivity to one of these clusters located in gag p17 was inversely correlated with viral load set point in an independent cohort of non-controllers.

**Conclusions:**

Antibody reactivity was low in non-controllers suppressed on ART, but remained high in viremic controllers despite viral suppression. Antibodies in controllers and viremic non-controllers were directed against epitopes in diverse HIV proteins; higher reactivity against p17 peptides was associated with lower viral load set point. Further studies are needed to determine if these antibodies play a role in regulation of HIV viral load.

## Introduction

In the absence of antiretroviral treatment (ART), most persons living with HIV have on-going viral replication that leads to CD4+ T cell depletion and severe immunodeficiency. Effective ART suppresses viral replication to low levels which reduces morbidity, mortality and the risk HIV transmission (1). HIV controllers are individuals who control viral replication to low levels in the absence of ART (2). Elite controllers maintain viral loads of <50 copies/mL, while viremic controllers maintain viral loads of <2,000 copies/mL (3). These individuals usually maintain high CD4+ T cell counts for long periods before experiencing immune system decline (4, 5). In non-controllers, a viral load set point is usually established in the absence of treatment; higher viral load set points are associated with higher transmission rates and more rapid disease progression in the absence of treatment.

The mechanisms responsible for suppression of viral replication in HIV controllers are not well understood (4). Many elites and viremic controllers are infected with replication-competent virus (6–9), suggesting that host factors play a role in controlling viral replication. Class I major histocompatibility (MHC) alleles, such as HLA-B*57 and HLA-B*27, have been implicated in elite control through cohort studies (10) and genome-wide association studies (11, 12). This suggests a role for CD8+ T cells in HIV control. More effective CD8+ T cell responses have been observed in elite controllers compared to individuals with progressive HIV disease (13–16). Furthermore, *in vivo* depletion of CD8+ T cells in monkey elite controllers of simian immunodeficiency virus infection leads to loss of control of viral replication (17, 18). Other genetic studies suggest that certain natural killer cell receptors, such as the killer immunoglobulin-like receptors, may also be involved in elite control (19–21).

The role of humoral immunity in viremic control and control of viral load set point is not clear. Serologic assays used for cross-sectional HIV incidence estimation demonstrate that most elite controllers have lower antibody titers than viremic individuals (22). Broadly neutralizing antibodies are also more likely to be found in non-controllers with high viral loads (23). However, elite controllers do not have higher titers of neutralizing antibodies to autologous (24) or heterologous viruses (3, 25) compared to non-controllers. Some studies have suggested that some elite controllers may have higher levels of antibody-dependent cellular cytotoxicity (ADCC) than viremic persons (26), while other studies found that elite controllers do not have higher levels of either antibodies (27) or effector cells (28) that can mediate ADCC. A recent study suggested that while antibodies from elite controllers were not more effective for ADCC in any single assay, they were more likely to be polyfunctional than antibodies from non-controllers (29). However, it is not clear whether this polyfunctional antibody profile is a cause or consequence of elite control.

There are limited data comparing the fine specificity of anti-HIV antibodies in persons with suppressed viral load on ART and persons with low viral load set points. The VirScan assay is a highly multiplexed antibody profiling system that can be used to characterize the fine specificity of antibodies to viral peptides that lack highly conformational or glycosylated epitopes (30). We previously used VirScan to characterize the evolution of HIV antibodies in early- to late-stage HIV infection (31). In this report, we used VirScan to compare the antibody profiles in HIV controllers, non-controllers on ART, and viremic non-controllers, including persons with different viral load set points.

## Materials and Methods

### Samples Used for Analysis

Samples used to compare antibody reactivity in HIV controllers and non-controllers were grouped into two cohorts (**Table 1**). The Discovery Cohort included samples from elite controllers, viremic controllers, non-controllers suppressed on ART, and viremic non-controllers enrolled a clinic-based cohort [Study of the Consequences of the Protease Inhibitor Era (SCOPE) study], San Francisco, CA (32, 33). The Validation Cohort included samples from elite controllers enrolled in a clinic-based cohort [Johns Hopkins University (JHU) Elite Controller Cohort, Baltimore, MD (24)] and non-controllers from a clinic-based cohort who were virally suppressed on ART [JHU Moore Clinic (34), Baltimore, MD]. Samples used to analyze the association between viral load set point and antibody reactivity were obtained from a study of the natural history of HIV infection conducted in Uganda and Thailand [the Early Capture HIV Cohort Study (RV217 Cohort); this study recruited men and women at locations associated with transactional sex (35)]. Participants in the Discovery and Validation cohorts are from the United States, where most HIV strains are subtype B. The cohort used to evaluate antibody reactivity and viral load set point included samples from persons with diverse HIV subtypes and strains (**Table 1**).

**Table 1:** Samples used for analysis.

Study Cohort	Sample Source	Prevalent HIV Subtype	Participant Status	Viral load^a^ (copies/mL)	# persons	# samples
Discovery Cohort	SCOPE Study	B	Elite contollers	<40	13	13
			Viremic controllers	40-2,000	27	27
			Non-controllers suppressed on ART	<40	21	21
			Viremic non-controllers	>2,00	12	12
Validation Cohort	JHU Elite Controller Cohort^b^	B	Elite controllers	<50	29	29
	JHU Moore Clinic^c^	B	Non-controller suppressed on ART	<400	37	37
Analysis of viral load set point^e^	RV217 Cohort^d^	A, D, and other subtypes/strains	Longitudinal samples collected prior to ART initiation	Various	53	298

The table provides the sample source and type of samples included in the Discovery Cohort, Validation Cohort, and the longitudinal cohorts.

a Different cutoff for viral suppression were used in the four parent studies; an assay with a lower limit of quantification of 400 copies/mL was in the JHU Moore Clinic at the time of sample collection.

b Samples from the JHU Elite Controller Cohort were from the earliest available sample collection date.

c Samples from the JHU Moore Clinic were obtained from the first visit after ART initiation where the viral load was <400 copies/mL.

d The RV217 Cohort included participants from different countries and risk groups (29 Thailand, 15 Kenya, 9 Uganda; 25 cisgender women, 18 cisgender men, 10 transgender women; median age: 24 years, interquartile range [IQR]: 19-26; 2-7 samples per person, mean: 5.6 samples/person). Samples from this cohort were collected from 6 months after HIV seroconversion to the last visit prior to ART initiation. Seven participants had viral loads <2,000 copies/mL at all visits.

e Viral load set point for participants in the RV217 Cohort was determined using viral load values from 6 months after seroconversion to initiation of antiretroviral treatment or the onset of AIDS. These participants were infected with following HIV subtypes and strains: 23 AE, 11 A, 11 AD, 2 B, 1 C, 5 recombinant (3A/C, 1B/AE, 1 C/AE). ART, antiretroviral therapy; JH, Johns Hopkins.

### Informed Consent

The SCOPE, RV217 and JHU studies were approved by institutional review boards at the parent research institutions. Participants provided written informed consent for their samples to be used in HIV-related research.

### Antibody Profiling Using the VirScan Assay

Plasma samples were analyzed using the VirScan assay, as described previously (30, 31, 36). The VirScan library includes 3,384 HIV peptides spanning the viral genome, representing numerous HIV subtypes and strains (30). This includes 821 peptides in 21 proteins for subtype B; 203 peptides in 18 proteins in subtype A; and 224 peptides in 20 proteins in subtype D; the subtype/strain designation for other peptides is described previously (31). Peptides are expressed on bacteriophage from DNA tiles; the expressed peptides are 56 amino acids long with 28 amino acid overlaps. The number of peptides in each location varies across the HIV genome, reflecting the level of genetic diversity (31). The VirScan library also encodes peptides spanning the genomes of >200 other viruses that infect humans. Antibody reactivity to Ebola virus and rabies virus peptides was used to normalize HIV antibody binding data to account for differences in sequencing depth between samples (31). IgG concentrations in plasma samples were determined using an enzyme-linked immunosorbent assay (ELISA) for IgG, so that input to the VirScan assay was constant at 2 μg IgG per reaction (36). Data from mock immunoprecipitation reactions were used for normalization as described previously (31). After incubation with samples, antibody-bound phage was immunoprecipitated using magnetic beads coated with protein A and protein G. After bead washing, DNA tiles in the immunoprecipitated phage were amplified by polymerase chain reaction (PCR) using primers containing sample-specific barcodes. PCR products were sequenced using a NextSeq 500 instrument (Illumina, San Diego, CA) to determine the amino acid sequences of the antibody-bound peptides. The samples were tested *via* VirScan from May 2019 to August 2019.

### VirScan Data Analysis

Antibody reactivity to each peptide was quantified using normalized read counts and relative fold change values, as described (31). Briefly, a raw read count was obtained for each peptide, reflecting the number of times the corresponding DNA sequence was detected in immunoprecipitated phage. Raw read counts were log_10_ transformed after adding one read count to the result for each peptide in each sample. The log-transformed values for each peptide and each sample were then normalized to the average read count obtained for all Ebola virus and rabies virus peptides for the same sample, in order to adjust for sample-to-sample differences in sequencing depth (none of the participants had these infections and antibody reactivity to these viruses most likely reflected non-specific antibody binding or spurious cross reactivity). Finally, a log_10_ relative fold change value for each peptide was calculated by subtracting the median of the normalized values for the same peptide observed in mock immunoprecipitation reactions run on the same plate from the log_10_ normalized value of the peptide. Six to eight mock immunoprecipitations were included on each 96 well immunoprecipitation plate.

### Statistical Methods

For analysis of antibody reactivity in controllers *versus* non-controllers, statistical inference between groups was carried out by moderated t-tests (37). Multiple comparison corrected q-values (38) were calculated from the observed p-values. If a peptide had a q-value of 0.05, it was expected that 5% of the peptides with smaller p-values would be false positives. Peptides with a q-value <0.05 were considered to have significantly different levels of antibody reactivity in the two groups. To test for replication, permutation analysis was performed to compare antibody reactivity for a combined set of 62 pre-identified peptides in elite controllers *vs*. non-controllers. For this analysis, one-sided moderated t-tests were used to obtain a p-value for each of the 62 peptides. Fisher’s inverse chi-square test (39) was then used to calculate a test-statistic based on the full set of 62 p-values by adding the -log_10_ p-value for each peptide. Since the null distribution in Fisher’s test assumes independence of p-values, a permutation test with 10,000 permutations was used to assess statistical significance, accounting for dependence of test statistics (e.g., due to overlapping and homologous peptides). In each permutation, the group labels (29 elite controller labels, 37 non-controller labels) were randomly assigned to the 66 participants; a p-value for each of the 62 peptides was calculated using a one-sided moderated t-test statistic, and a test-statistic was obtained for the full set of peptides using Fisher’s method. Finally, a one-sided p-value of the permutation test was calculated as the proportion of sample permutations where the value of the test-statistic was greater than or equal to the observed test-statistic.

For analysis of viral load set point, the set point for each individual (log_10_ HIV RNA copies/mL) was calculated by taking the median pre-ART viral load for each person. Median antibody reactivity (log_10_ normalized fold change) was calculated for the same samples. Simple linear regression was conducted for each peptide with viral load set point as the dependent variable and median antibody reactivity as the independent variable. The estimate of the slope is reported as effect size; the statistical significance of the effect size (p-value) was derived by testing the hypothesis that the true slope was zero (i.e., no association between viral load set point and antibody reactivity). Multiple comparisons correction was carried out by controlling the family-wise error rate using the Bonferroni method. Due to the homology of peptides in the VirScan library and possible dependence of test statistics and p-values, a permutation test was also performed, jointly shuffling the viral load set point across all peptides, thereby maintaining the correlation structure.

The software environment R (version 4.0.1) was used for statistical computing. The software epitopefindr (GitHub: https://brandonsie.github.io/epitopefindr/) was used to identify amino acid motifs associated each peptide clusters.

## Results

### Comparison of Antibody Profiles in HIV Controllers and Non-Controllers

**Figure 1:**
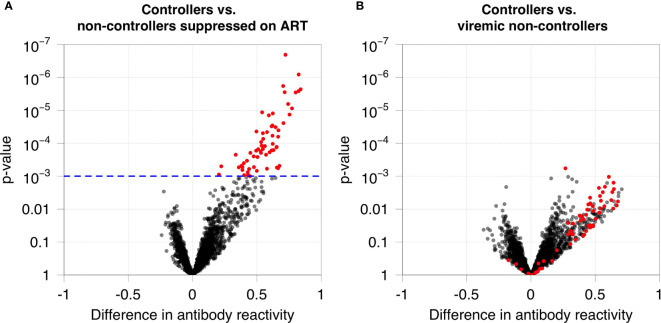
Antibody reactivities in HIV controllers compared to non-controllers. **(A)** Antibody reactivity was compared for 40 HIV controllers (13 elite controllers and 27 viremic controllers) and 21 non-controllers suppressed on ART. The volcano plot shows on the x-axis the difference in antibody reactivity in the two groups (estimated log_10_ fold change; positive numbers correspond to stronger reactivity in controllers) and the y-axis shows the -log_10_ p-value for each peptide based on moderated t-statistics. Red dots indicate the 62 peptides that are significantly more reactive in controllers at a false discovery rate of 5%. The blue dashed line indicates the highest q-value less than 5% (q=0.0485), which corresponds to a p-value of 0.001. **(B)** Antibody reactivity to each HIV peptide was compared for 40 HIV controllers and 12 viremic non-controllers. No significant differences in antibody reactivity were observed for these 62 peptides in these two groups.

We first used VirScan to compare antibody reactivity among 40 HIV controllers and 33 non-controllers in the Discovery Cohort (**Table 1**). Antibody reactivity was quantified for each peptide in the VirScan assay. Sixty-two peptides had significantly higher levels of antibody reactivity in HIV controllers compared to non-controllers who were virally suppressed on ART (**Figure 1A**). Despite observing a similar trend for most of these peptides when comparing HIV controllers to viremic non-controllers none of the peptides displayed statistically significant differences in reactivity in these two groups after multiple testing correction (**Figure 1B**).

**Figure 2:**
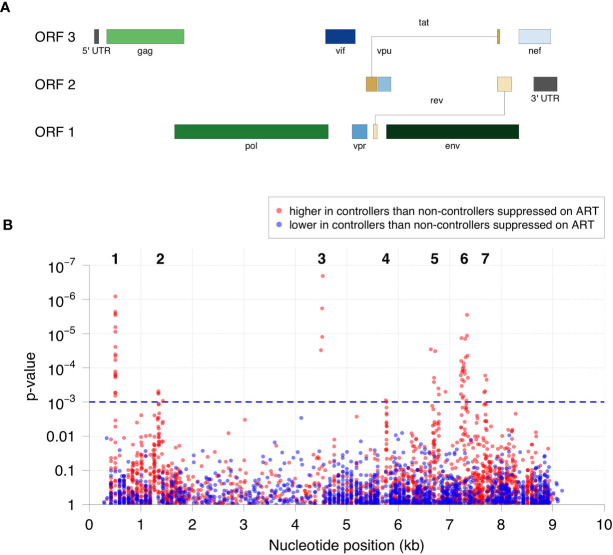
Specificity of antibodies targeted in HIV controllers compared to non-controllers who were virally suppressed on ART. **(A)** The positions and lengths of open reading frames (ORFs) in the HIV genome are plotted relative to genomic coordinates for HIV (HXB2, NCBI #NC_001802). **(B)** The significance for the difference in antibody reactivity in controllers *versus* non-controllers who were virally suppressed on ART is shown for each peptide. The x-axis shows the position of each peptide in the HIV genome. The y-axis shows the -log_10_ p-value based on moderated t-statistics for each peptide. Each dot shows the result for a single peptide. Red dots indicate peptides that had higher antibody reactivity in the HIV controller group. Blue dots indicate peptides that had lower antibody reactivity in the HIV controller group. The blue dashed line indicates the highest q-value less than 5% (q=0.0485), which corresponds to a p-value of 0.001. The 62 significant peptides with q-values <0.05 (above the blue dashed line) had significantly higher peptide reactivity in HIV controllers compared to non-controllers who were virally suppressed on ART. Cluster numbers (1-7) are noted above each group of clustered peptides (see **Table 2**).

The relative level of antibody reactivity to peptides in HIV controllers *versus* non-controllers who were virally suppressed on ART varied as a function of peptide location (**Figures 2**, **S1**). Sixty-one of the 62 peptides formed seven distinct clusters of homologous peptides (**Figure 2B** and **Table S1**). Twenty-two peptides were located in the gag region: 18 in the N-terminus of gag (p17, cluster 1) and four in the C-terminus of gag (p24, cluster 2). Four peptides were located in the C-terminus of integrase (cluster 3). Two peptides were located in the N-terminus of the vpu accessory protein (cluster 4). The remaining 34 peptides were located in the env region. Seven of these peptides spanned the V3 loop and CD4 binding loop of gp120 (six in cluster 5, one that was not in a cluster), 23 spanned the V5 loop of gp120 and the fusion peptide of gp41 (cluster 6), and four were in gp41, proximal to the membrane proximal external region (MPER, cluster 7). The peptides detected in each cluster are provided in **Table 2**.

**Table 2:** Peptides with higher antibody reactivity in HIV controllers compared to non-controllers who were virally suppressed on antiretroviral therapy.

Peak	Genomic Location	HXB2 Coordinates	Motif	# peptides	Sequence Logo
1	N-terminus of gag	420-588	1	18	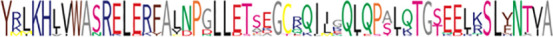
2	C-terminus p24	1251-1512	2	4	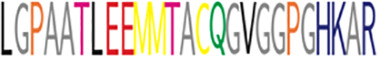
3	C-terminus of integrase	4415-4616	3	4	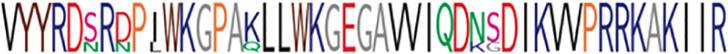
4	N-terminus of the cytoplasmic domain of vpu	5674-5854	4	2	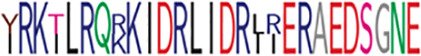
5	gp120, spanning V3 and the CD4 binding loop	6548-6872	5a	4	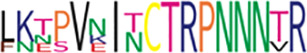
			5b	6	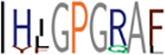
6	gp120/41, spanning V5 and the fusion peptide	7130-7436	6a	23	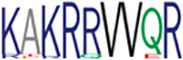
			6b	21	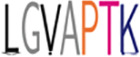
7	gp41, HR2 domain	7586-7799	7	4	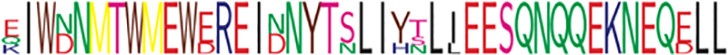

The table shows features of the 62 peptides that had higher antibody reactivity in HIV controllers compared to non-controllers who were virally suppressed on ART. The 62 peptides were located in seven clusters. HIV protein location was determined using the full-length peptides in each cluster. HXB2 coordinates are shown for each cluster (HXB2, NCBI #NC_001802). The number of potential peptide targets in the VirScan library varied from region to region, reflecting the level of viral diversity; the library included 35 peptides in cluster 1; 60 peptides in cluster 2; 5 peptides in cluster 3; 32 peptides in cluster 4; 593 peptides in cluster 5; 77 peptides in cluster 6; and 21 peptides in cluster 7.

**Figure 3:**
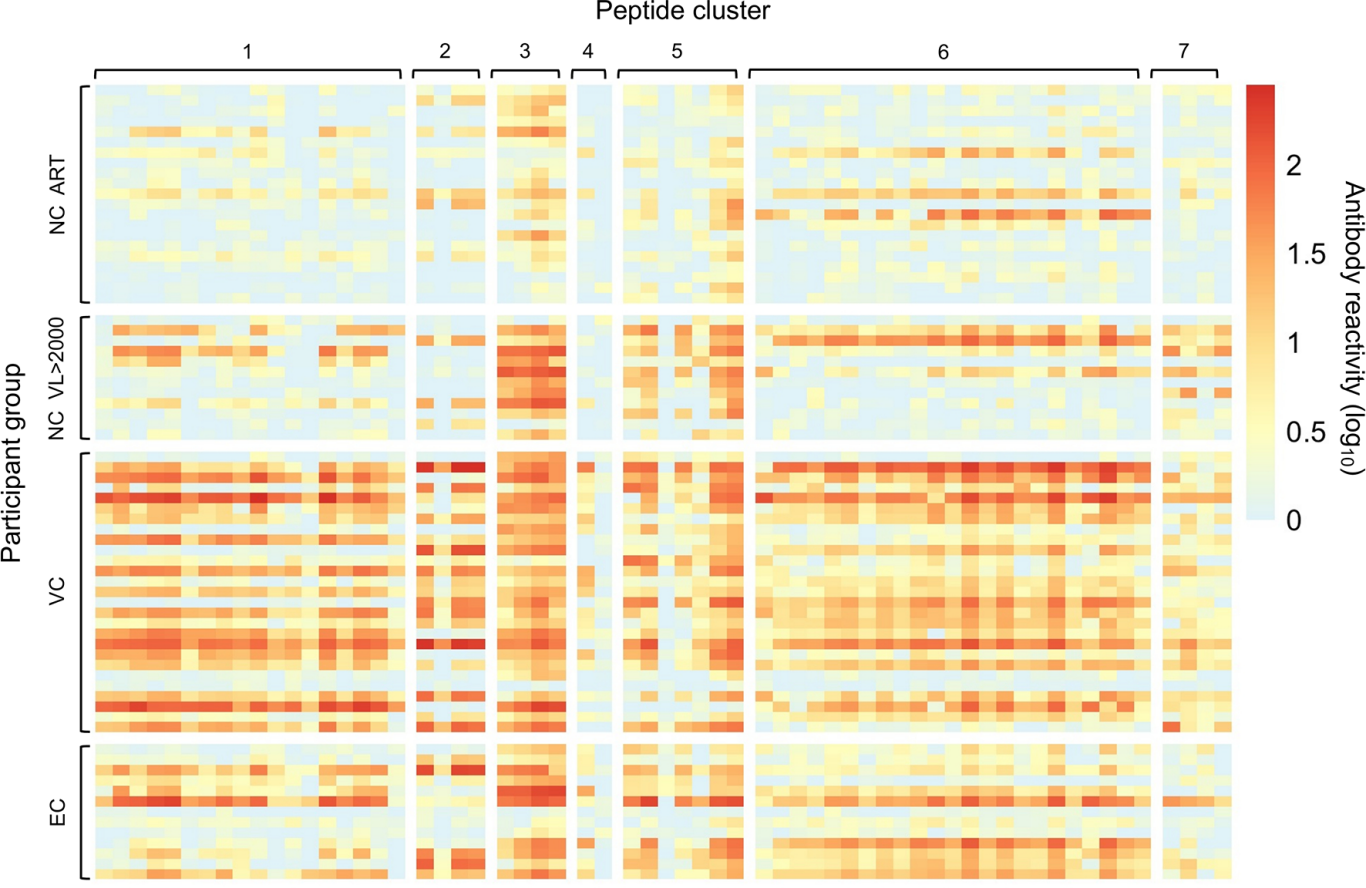
Individual patterns of antibody reactivity to peptides in peptide clusters. The patterns of antibody reactivity are shown for the 13 individual elite controllers (EC), 27 viremic controllers (VC), 12 non-controllers with viral loads >2,000 copies/mL (NC VL >2000), and non-controllers who were virally suppressed on ART (NC ART). Colors indicate the level of antibody reactivity (log_10_ fold change in VirScan read counts compared to mock immunoprecipitation reactions); values less than zero were assigned a value of zero. Each colored square represents data for a single peptide from a single individual. Darker colors indicate higher levels of antibody reactivity. Data are grouped by participant group and peptide cluster (see **Table 2**).

The program epitopefindr (40) was used to identify amino acid motifs that were common to peptides in each cluster. The number of peptides included in each motif varied from two to 23 (**Table 2**). Five clusters had one motif (clusters 1-4 and 7) and two had two motifs (clusters 5 and 6). **Table 2** shows additional characteristics of the nine motifs. Note that one peptide near cluster 5 did not include either of the motifs associated with other peptides in that cluster. The pattern of antibody reactivity to peptides in each cluster varied among study participants (**Figure 3**). Most elite and viremic controllers had increased antibody reactivity to multiple peptides in multiple clusters. In contrast, antibody reactivity was minimal among non-controllers who were virally suppressed on ART and was predominantly observed for integrase peptides in viremic non-controllers (cluster 3).

### Analysis of Antibody Reactivity in Independent Cohorts

We next evaluated antibody reactivity to the 62 significant peptides using samples from an independent Validation Cohort that included samples from 29 elite controllers and 37 non-controllers who were virally-suppressed on ART (**Table 1**). **Figure S2** shows the difference in antibody reactivity in these two groups. Reactivity in elite controllers was higher for 51 peptides and lower for 11 peptides. We used a permutation test to compare antibody reactivity for all 62 peptides combined in the two participant groups. The average antibody reactivity for the combined set of peptides was significantly higher for elite controllers than for virally suppressed non-controllers (p=0.024).

### Association of HIV Peptide Reactivity With the Protective HLA-B*57 Allele

We then evaluated the association between antibody reactivity and the presence of the protective HLA-B*57 allele. This analysis was performed using samples from the 40 HIV controllers in the Discovery Cohort (21) (27 with the HLA-B*57 allele, 13 without the allele; **Figure S3**). None of the peptides in the VirScan library (including the 62 peptides described above) had antibody reactivity that was significantly different between these two groups.

### Association Between Viral Load Set Point and Antibody Reactivity

**Figure 4:**
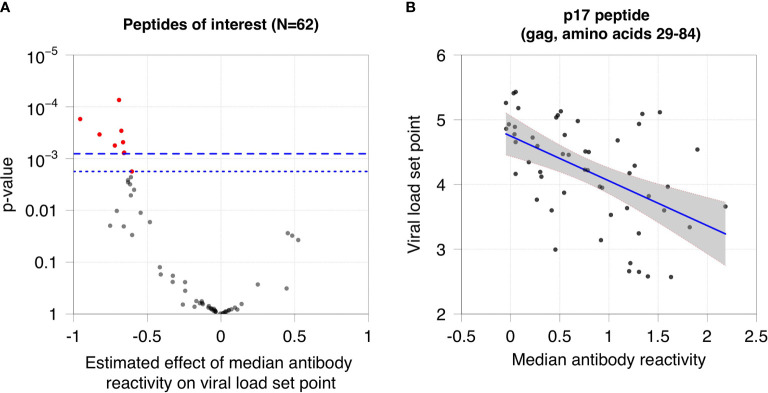
Association between viral load set point and antibody reactivity in the RV217 Cohort. **(A)** The plot shows the association between viral load set point and median antibody reactivity for the 62 peptides of interest. The x-axis shows the estimated effect of median antibody reactivity on viral load set point (effect size from the regression analysis); the y-axis shows the -log_10_ p-value of the association. Negative effect sizes indicate that antibody reactivity was higher for participants with lower viral load set points. The dashed line indicates the cutoff for significance using the Bonferroni correction (p=0.0008); the dotted line indicates the cutoff for significance using a permutation test (p=0.0018). Red dots indicate peptides that had a statistically significant association between viral load set point and median antibody reactivity. **(B)** The plot shows the median reactivity of the peptide (x-axis) that had the strongest observed association with viral load set point (y-axis). Each dot represents data from a single participant. The blue line indicates the least squares regression line. The grey shaded area represents the 95% confidence band for the mean viral load set point.

We next evaluated whether antibody reactivity to the 62 peptides was associated with viral load set point in non-controllers who were not on ART. This analysis was performed using samples from the RV217 Cohort, which included men and women from East Africa and Thailand with different risk factors for infection. Antibody reactivity to nine of the 62 peptides was significantly associated with viral load set point after using a permutation test for multiple testing correction (**Figure 4**). All nine peptides were located in gag protein, p17 (cluster 1; **Table S2**).

## Discussion

Using the VirScan antibody profiling assay, we compared the antibody profiles in viremic non-controllers to persons with natural HIV control and those who were virally suppressed on ART. In a previous study, we demonstrated that most persons living with HIV express a diverse array of anti-HIV antibodies (31). This report was focused on characterization of antibodies in persons with natural and ART-induced viral suppression and persons with lower viral load set points. Previous studies show that overall HIV antibody expression is down-regulated in HIV controllers and individuals who are virally suppressed on ART (22, 41).

We identified groups of peptides that were preferentially targeted by antibodies in HIV controllers and persons with lower viral load set points. These peptides were grouped into homologous clusters in HIV env (gp120 and gp41), gag (p17 and p24), integrase, and vpu. Most of the HIV controllers in the Discovery Cohort had antibodies to peptides in all or most of the seven clusters. The level of reactivity to these peptides was similar in viremic non-controllers and HIV controllers, but was significantly lower in non-controllers who were suppressed on ART. Aggregate antibody reactivity to the same peptides was significantly higher in elite controllers compared to ART-suppressed non-controllers in an independent cohort. These findings demonstrate that elite and viremic HIV controllers produce many of the same antibodies as viremic non-controllers, and that these antibodies persist in HIV controllers despite long-term, natural suppression of viral replication. It is not clear whether the high levels of expression of these antibodies in HIV controllers reflects on-going viral replication (antigen stimulation) or other factors. It is also not clear whether these antibodies play a role in the natural control of HIV infection.

We also found that the pattern of antibody reactivity to peptides in the clusters varied among non-controllers, depending on HIV viral load. In the RV217 Cohort, antibody reactivity to peptides in the p17 cluster was significantly higher in non-controllers with lower viral load set points. These peptides are located in the p17 matrix protein. p17 is released from infected cells and plays a critical role in the viral replication cycle (42). This protein exerts cytokine-like activities that promote viral replication through interaction with target cell receptors (43). The functional epitope of p17 is located at the N-terminus of the protein (44), immediately upstream from the peptides identified in this report. Antibodies to p17 are associated with slower HIV disease progression (45). A synthetic peptide that includes the functional epitope of p17 was evaluated in a Phase 1 clinical trial as a therapeutic vaccine (46, 47). Further studies are needed to determine whether antibodies directed against p17 or other gag epitopes identified in this study play a functional role in natural control of HIV replication.

Three of the seven peptide clusters identified in this study were in HIV env. The capacity of env antibodies to modulate HIV replication is well-established. Broadly neutralizing monoclonal antibodies directed against HIV env are being evaluated in clinical trials as interventions for HIV prevention and treatment (48). These antibodies target epitopes on the surface of gp120 (e.g., in the CD4 binding site, the V3 loop, and V1/V2 region) or gp41 (in the MPER) (48). Most of the env peptides identified in this report were in gp120, spanning the V3 loop and CD4 binding loop or the V5 loop and fusion peptide; four peptides were located in the coiled coil domain of gp41, proximal to the MPER.

Antibodies to integrase peptides were also targeted by HIV controllers in this report. These antibodies were also observed in some viremic non-controllers; however, this reactivity was much lower when non-controllers were virally suppressed on ART. Antibody reactivity to non-surface proteins has been observed in other viral infections. For example, strong reactivity to nuclear antigens is observed in Epstein Barr virus infection (49), and reactivity to the nucleocapsid protein is often used to evaluate prior SARS-CoV-2 infection (50). In HIV-infected individuals, integrase antibodies often appear after antibodies to other HIV targets (51, 52). Intracellular antibody fragments directed against HIV integrase or its cellular target have been shown to inhibit viral replication (53, 54).

We also identified vpu peptides that were preferentially targeted in HIV controllers compared to non-controllers who were suppressed on ART. Vpu is a transmembrane protein that enhances virion release from infected cells by antagonizing the host restriction factor, tetherin. Enhanced release of virions by vpu may reduce antibody binding to infected cells by limiting the amount of time that gp120 is expressed on the cell surface (55). In a previous study, antibodies directed against specific ADCC vpu epitopes were detected in long-term slow progressors (LTSP), but not in a control group of non-LTSP (56). Antibodies directed against a vpu peptide were also more likely to elicit responses in an ADCC assay in elite controllers compared to viremic individuals (57). The vpu peptides targeted by LTSP and elite controllers in those studies overlap with the vpu peptides that were preferentially targeted in HIV controllers in this report. The sequence of one peptide cluster from the LTSP study was contained within the vpu amino acid motif identified in this report; the sequence of another peptide cluster from the LTSP study overlapped with that motif (**Table S3**).

Many assays used to evaluate the specificity of HIV antibodies include antigen targets that are expressed as proteins rather than discrete peptides, or include a small number of protein and peptide targets (58–60). In contrast, the VirScan assay provides a comprehensive, unbiased platform for assessing the full range of linear, non-glycosylated HIV peptide targets. By aligning reactive peptides that share sequence homology, VirScan also provides information on the fine specificity of antibodies, including specific amino acid motifs that were preferentially targeted in HIV controllers. Further studies are needed to characterize antibody binding to non-linear and glycosylated epitopes.

Several factors may have limited our ability to identify the full range of antibody targets associated with viremic control of HIV infection. The sample sets used for peptide discovery were relatively small and were limited to samples from the United States that were likely to have subtype B HIV. These samples were collected from the general population and from a cohort comprised predominantly of men who have sex with men; other HIV subtypes and risk groups were not represented. Another limitation of this study is that the VirScan assay only detects antibody reactivity to linear epitopes in unglycosylated target peptides; the conformation of peptides expressed on bacteriophage may also differ from the natural conformation of peptides expressed *in vivo*. Antibodies to peptides in the HIV antisense protein ^62^ will also not be detected, since the current VirScan library does not include these peptides. This study also does not provide any information about cellular responses to HIV infection, which have been shown to play an important role in viremic control of HIV infection. However, T cell and B cell responses are coordinated and frequently target closely linked or overlapping epitopes. The antibody specificities reported here may therefore be associated with enhanced T cell recognition of linked epitopes presented on infected cells.

## Conclusion

The studies cited above and findings in this report demonstrate that expression of antibodies against gp120, gp41, p17, integrase, and vpu persist in HIV controllers despite viral suppression. Additional research is needed to determine whether antibodies directed against peptides in these regions play a role in controlling HIV replication, and/or are indicators of enhanced T cell responses. This could be evaluated by comparing proviral and plasma sequences from HIV controllers to assess whether there is evidence of antibody-mediated selection against these peptides; this approach was used previously to identify cytotoxic T lymphocyte (CTL) escape mutations in HIV controllers reflecting selective pressure against HLA-B*57-restricted epitopes (61). It may also be possible to generate antibodies against epitopes identified in this report, or to isolate those antibodies from HIV controllers, and to test whether those antibodies can neutralize HIV or exert other functional effects on viral replication. Further characterization of antibodies in HIV controllers in other cohorts and populations may provide further insights into antibody-mediated control of HIV infection. This information may help inform development of therapeutic vaccines and monoclonal antibodies for HIV treatment and prevention. The VirScan assay also contains phage that express peptides spanning the full genomes of >200 other viruses that infect humans. The approach used in this report could also be used to identify peptides associated with control of viral replication for other clinically relevant viruses.

## Data Availability Statement

The raw data supporting the conclusions of this article will be made available by the authors, without undue reservation.

## Ethics Statement

The studies involving human participants were reviewed and approved by IRB. The patients/participants provided their written informed consent to participate in this study.

## Author Contributions

HBL: Designed the study, analyzed data, and drafted the manuscript. SE: Designed the study, analyzed data, and drafted the manuscript. OL: Designed the study and provided input into data analysis. DM: Performed testing and analyzed data. AC: Contributed to study design and analyzed data. SH: Performed testing. WG-M: Assisted with literature review. GA: Provided samples and data from the SCOPE Study. RDM: Project lead for the JHU Moore Clinic Cohort. SD: Project lead for the SCOPE Study. LE: Investigator for the RV217 Study. CM: Project lead for the Genital Shedding Study. JB: Provided expertise on elite and viremic HIV controllers. IR: Provided statistical and bioinformatics expertise and analyzed data. KK: Provided statistical and bioinformatics expertise, analyzed data, and drafted the manuscript. All authors contributed to the article and approved the submitted version.

## Funding

This work was supported by the National Institute of Allergy and Infectious Diseases (NIAID) of the National Institutes of Health (NIH) through R01-AI095068 and the National Institute of General Medical Sciences (NIGMS) through R01-GM136724. Additional support was provided through the Laboratory Center of HIV Prevention Trials Network (HPTN) which is sponsored by the NIAID, National Institute on Drug Abuse, National Institute of Mental Health, and Office of AIDS Research, of the NIH, DHHS (UM1-AI068613), and through intramural funding from the Division of Intramural Research, NIAID, NIH. The Johns Hopkins HIV Cohort was supported by NIH grants U01-DA036935 and U01-AI069918. The Johns Hopkins Medicine Elite Controller Cohort was supported by NIH grant R01-AI140789. The RV217 study was supported by a cooperative agreement (W81XWH-18-2-0040) between the Henry M. Jackson Foundation for the Advancement of Military Medicine, Inc., and the U.S. Department of Defense. This research was also funded, in part, by NIAID. The SCOPE cohort was supported by the UCSF/Gladstone Institute of Virology & Immunology CFAR (P30-AI027763).

## Author Disclaimer

The views expressed in this report are those of the authors and should not be construed to represent the positions of the U.S. Army, the Department of Defense, or the Henry M. Jackson Foundation. The investigators in the RV217 Study adhered to the policies for protection of human subjects as prescribed in the Army Publishing Directorate AR 70–25.

## Conflict of Interest

HBL is an inventor on a patent application (US20160320406A) filed by Brigham and Women’s Hospital that covers the use of the VirScan technology to identify pathogen antibodies and is a founder of ImmuneID, Portal Bioscience and Alchemab, and is an advisor to TScan Therapeutics.

The remaining authors declare that the research was conducted in the absence of any commercial or financial relationships that could be construed as a potential conflict of interest.

## Publisher’s Note

All claims expressed in this article are solely those of the authors and do not necessarily represent those of their affiliated organizations, or those of the publisher, the editors and the reviewers. Any product that may be evaluated in this article, or claim that may be made by its manufacturer, is not guaranteed or endorsed by the publisher.
