# Supplementary material for: HIV Antibody Profiles in HIV Controllers and Persons With Treatment-Induced Viral Suppression
Source: Front Immunol. 2021 Aug 26;12:740395. doi: 10.3389/fimmu.2021.740395 (PMC8428532; doi:10.3389/fimmu.2021.740395)
Supplement: Supplementary file 1 [file DataSheet_1.pdf]

**Supplementary Materials**

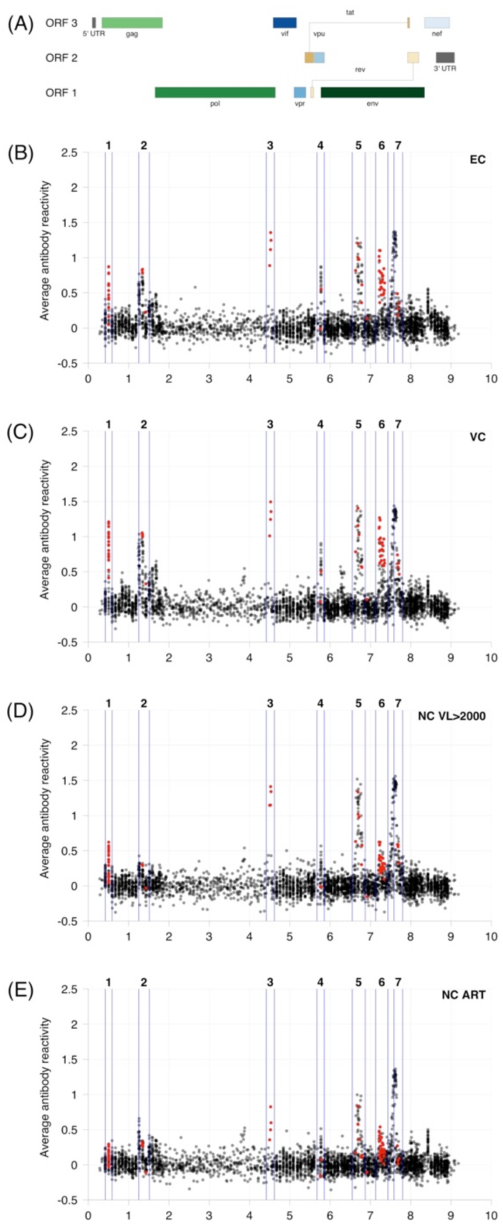

**Figure S1. Antibody reactivity in the Discovery Cohort. (A)** The positions and lengths of open reading frames (ORFs) in the HIV genome are plotted relative to genomic coordinates for HIV (HXB2, NCBI #NC_001802). **(B)-(E)** Data from the Discovery Cohort (SCOPE Study), including 13 elite controllers (EC), 27 viremic controllers (VC), 12 non-controllers with viral loads >2,000 copies/mL (NC VL >2000), and non-controllers who were virally suppressed on ART (NC ART). In each panel **(B)-(E**), the x-axis shows the position of each peptide in the HIV genome and the y-axis shows the average antibody reactivity ($\log_{10}$ normalized fold change) for each HIV peptide. Red dots indicate the 62 peptides that had significantly higher reactivity in HIV controllers compared to non-controllers who were virally suppressed on antiretroviral therapy (see **Figure 2**). Peptides are assigned HXB2 coordinates based on the position of the middle amino acid in each peptide. The genomic locations of the seven clusters described in **Table 2** are indicated by vertical lines (cluster 1: p17; cluster 2: p24, cluster 3: integrase; cluster 4: vpu; clusters 5 and 6: gp120; cluster 7: gp41; see **Table S1**).

**Supplementary Materials**

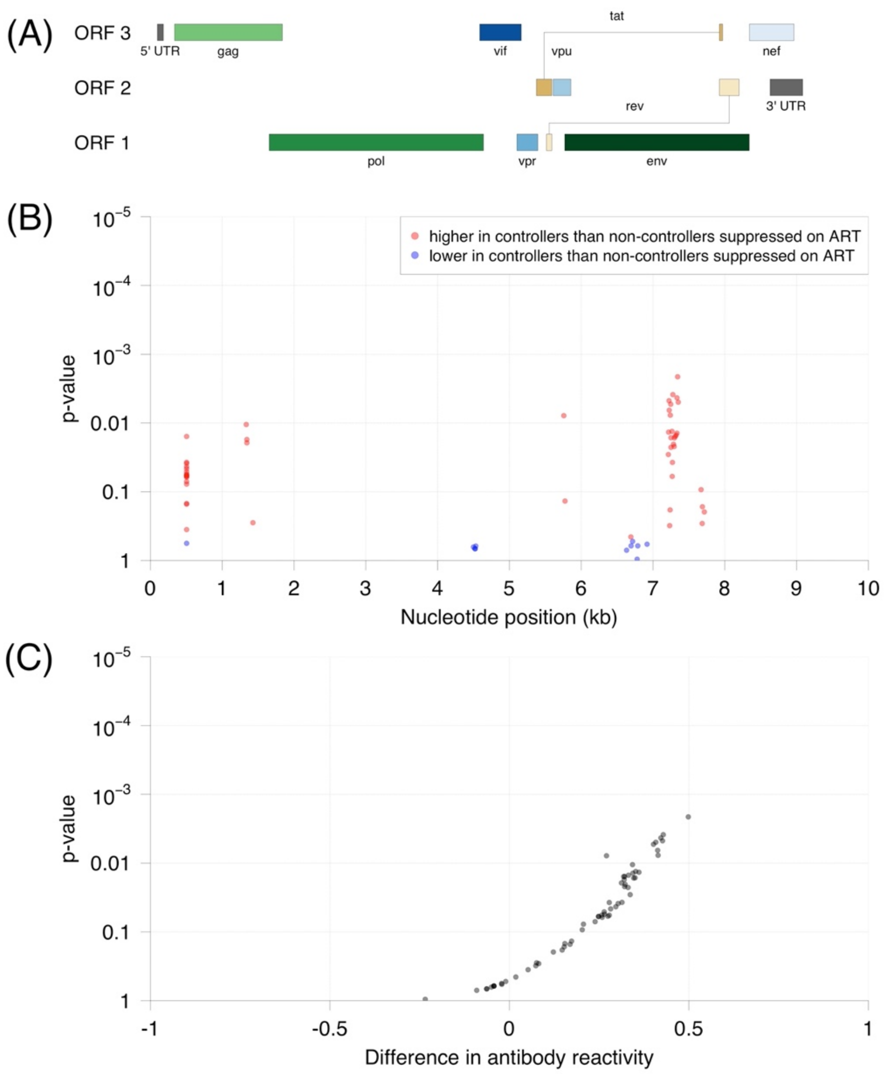


**Figure S2: Comparison of antibody reactivity for 62 significant peptides in elite controllers compared to non-controllers who were virally suppressed on antiretroviral therapy (Validation Cohort).** In the Discovery Cohort, 62 peptides were identified that had significantly higher antibody reactivity in HIV controllers compared to non-controllers who were virally suppressed on antiretroviral therapy (ART) (**Figure 2**). This figure shows antibody reactivity to the 62 significant peptides in 29 elite controllers and 37 non-controllers who were virally suppressed on ART in the independent Validation Cohort. **(A)** The figure shows the positions and lengths of open reading frames (ORFs) in the HIV genome plotted relative to genomic coordinates for HIV (HXB2, NCBI #NC_001802). **(B)** The plot shows the significance for the difference in antibody reactivity for each of the 62 peptides in the two participant groups. The x-axis shows the position of each peptide in the HIV genome. The y-axis shows the -$\log_{10}$ p-value based on moderated t-statistics for each peptide. Each dot represents the result obtained for a single peptide. Red dots indicate peptides that had higher antibody reactivity in the elite controller group. Blue dots indicate peptides that had lower antibody reactivity in the elite controller group. **(C)** In the volcano plot, the x-axis shows the difference in antibody reactivity in the two participant groups (estimated $\log_{10}$ fold change) and the y-axis shows the -$\log_{10}$ p-value for a one-sided comparison for each peptide based on moderated t-statistics. Each dot represents data for a single peptide.

## Supplementary Materials

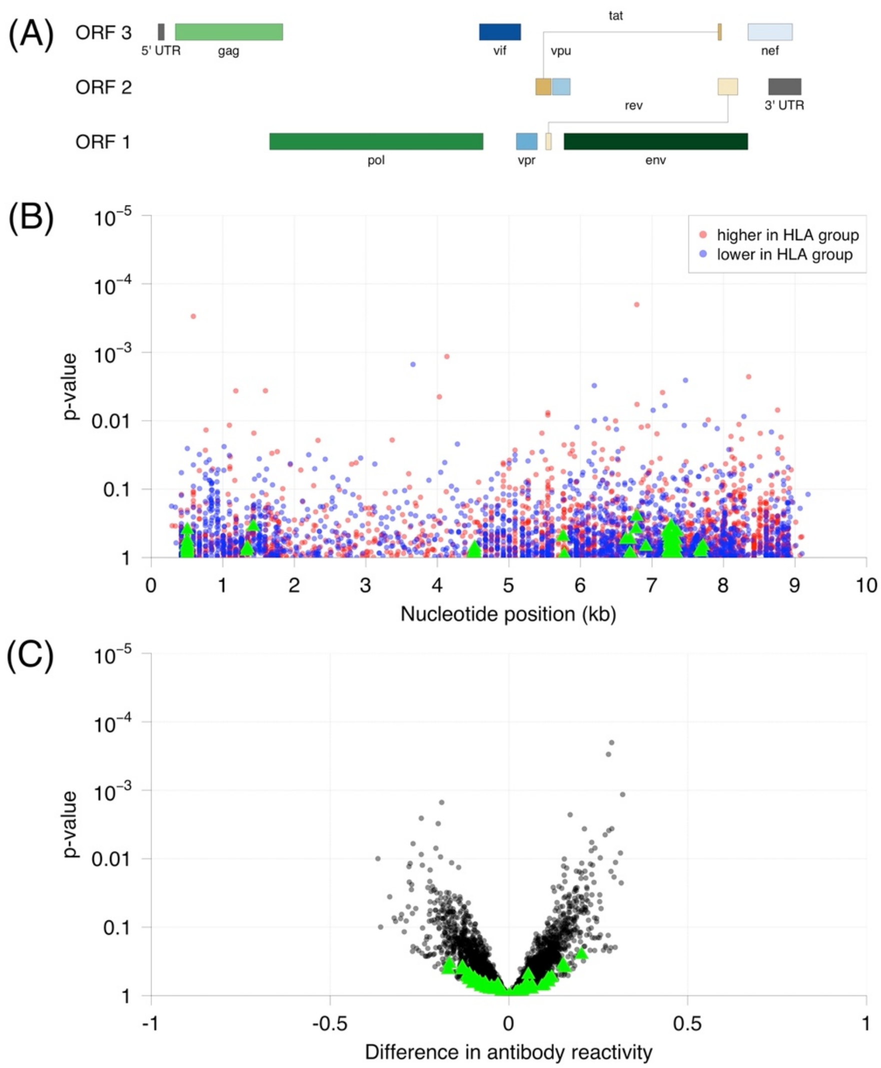


**Figure S3. Association of antibody reactivity and the presence of the HLA-B*57 allele in HIV controllers (Discovery Cohort).** Antibody reactivity to each HIV peptide was compared for 27 HIV controllers with HLA-B*57 allele and 13 HIV controllers without the HLA-B*57 allele (Discovery Cohort) using moderated t-tests. **(A)** The positions and lengths of open reading frames (ORFs) in the HIV genome are shown relative to genomic coordinates for HIV (HXB2, NCBI #NC_001802). (**B)** Red dots indicate peptides that had higher antibody reactivity in HIV controllers with the HLA-B*57 allele. Blue dots indicate peptides that had lower antibody reactivity in HIV controllers with the HLA-B*57 allele. Data for the 62 peptides that had significantly higher reactivity in HIV controllers compared to non-controllers who were virally suppressed on antiretroviral therapy (ART, q-values <0.05) are shown as green triangles. **(C)** In the volcano plot, the x-axis shows the difference in antibody reactivity between the two participant groups (estimated $\log_{10}$ fold change) and the y-axis shows the $-\log_{10}$ p-value for each peptide based on moderated t-statistics. Each dot represents data for a single peptide. At a false discovery rate of 5%, none of the peptides had antibody reactivity that was significantly different among HIV controllers with the HLA-B*57 allele and those without the allele. Green triangles indicate the 62 peptides that had significantly higher reactivity in HIV controllers compared to non-controllers who were virally suppressed on ART.

## Supplementary Materials

**Table S1. Characteristics of the 62 peptides that had significantly higher antibody reactivity in HIV controllers compared to non-controllers who were virally suppressed on antiretroviral therapy (Discovery Cohort).** The table shows the characteristics of the 62 peptides that had significantly higher antibody reactivity in HIV controllers compared to non-controllers who were virally suppressed on ART (Discovery Cohort). The following information is provided for each of the 62 peptides: Peptide identifier; HIV gene location; HIV protein location; amino acid position; HXB2 coordinates (HXB2, NCBI #NC_001802); amino acid sequence; UniProt number; cluster designation (see Figure 2 and Table 2).

[a] Peptides in the C-terminus of p24 do not include spacer peptide 1.

[b] Peptides in the C-terminus of integrase are located C-terminal to the catalytic domain.

[c] Peptides in the gp41 coiled coil domain are just N-terminal to the membrane-proximal external region (MPER).

| Peptide ID | HIV gene location | HIV protein location | Amino acid position | HXB2 coordinates | Amino acid sequence | UniProt number | Cluster |
|---|---|---|---|---|---|---|---|
| pep17419 | gag | N-terminus of gag (p17) | 29-84 | 420-588 | YMMKHLVWASRELERFALDPGLLETSEGCKQIMKQLQPALQTGTKELISLHNTVAT | O12157 | 1 |
| pep19339 | gag | N-terminus of gag (p17) | 29-84 | 420-588 | YRLKHLVWASRELERFALDPGLLETSEGCRKIIGQLQPSLQTGSEELKSLYNTIAV | O89291 | 1 |
| pep20344 | gag | N-terminus of gag (p17) | 29-84 | 420-588 | YRLKHLVWASRELERFALNPGLLETPEGCLQIIEQIQPAIKTGTEELKSLFNLVAV | O93182 | 1 |
| pep21855 | gag | N-terminus of gag (p17) | 29-84 | 420-588 | YKLKHIVWASRELERFAVNPGLLETSEGCRQILGQLQPSLQTGSEELRSLYNTVAT | P03347 | 1 |
| pep22039 | gag | N-terminus of gag (p17) | 29-84 | 420-588 | YKLKHIVWASRELERFAVNPGLLETSEGCRQILGQLQPSLQTGSEELRSLYNTVAT | P03366 | 1 |
| pep23225 | gag | N-terminus of gag (p17) | 29-84 | 420-588 | YRLKHLVWASRELERFALNPGLLETGEGCQQIMEQLQSTLKTGSEEIKSLYNTVAT | P04588 | 1 |
| pep23294 | gag | N-terminus of gag (p17) | 29-84 | 420-588 | YRLKHIVWASRELERYALNPGLLETSEGCKQIIGQLQPAIQTGTEELRSLYNTVAT | P04592 | 1 |
| pep24066 | gag | N-terminus of gag (p17) | 29-84 | 420-588 | YKLKHVVWASRELERFAINPGLLETSEGCRQILGQLQPSLQTGSEERKSLYNTVAT | P05888 | 1 |
| pep24143 | gag | N-terminus of gag (p17) | 29-84 | 420-588 | YRLKHIVWASRKLERFAVNPGLLETSKGCRQILGQLQPSLQTGSEELRSLYNTVAT | P05960 | 1 |
| pep31689 | gag | N-terminus of gag (p17) | 29-84 | 420-588 | YRLKHLVWASRELERFALNPGLLETSDGCKQIIGQLQPAIRTGSEELRSLFNTVAT | P12495 | 1 |
| pep64581 | gag | N-terminus of gag (p17) | 29-84 | 420-588 | YMLKHLVWANRELEKFALNPDLLDTSAGCKQIIKQLQPALQTGTEELKSLFNTVAT | Q75001 | 1 |
| pep64598 | gag | N-terminus of gag (p17) | 29-84 | 420-588 | YMLKHLVWANRELEKFALNPDLLDTSAGCKQIIKQLQPALQTGTEELKSLFNTVAT | Q75002 | 1 |
| pep65516 | gag | N-terminus of gag (p17) | 29-84 | 420-588 | YRLKHLVWASRELERYACNPGLLETAEGTEQLLQQLEPALKTGSEDLKSLWNAIAV | Q79665 | 1 |
| pep76931 | gag | N-terminus of gag (p17) | 29-84 | 420-588 | YRLKHLVWASRELERFALNPDLLETADGCQQILGQLQPALKTGTEDLQSLYNTIAV | Q9Q721 | 1 |
| pep77261 | gag | N-terminus of gag (p17) | 29-84 | 420-588 | YKLKHLVWASRELERFALNPGLLETTEGCRQIITQIQPSIQTGSEEIKSLYNTIAV | Q9QBY4 | 1 |
| pep77406 | gag | N-terminus of gag (p17) | 29-84 | 420-588 | YRLKHLVWASRELERFALNPNLLETVEGCRQIIRQLQPSLQTGSEELRSLFNTVAT | Q9QC00 | 1 |
| pep78255 | gag | N-terminus of gag (p17) | 29-84 | 420-588 | YKMKHLIWASRELERFALDPGLLETSEGCQKIIRQLQPSLQTGSEELKSLFNTVAV | Q9QSR3 | 1 |
| pep78360 | gag | N-terminus of gag (p17) | 29-84 | 420-588 | YRIKHLVWASRELDRFALNPGLLESAKGCQQILVQLQPALQTGTQEIKSLYNTVAT | Q9WC53 | 1 |
| pep24076 | gag | C-terminus of p24[a] | 309-364 | 1251-1419 | AEQASQEVKNWMTETLLVQNANPDCKTILKALGPAATLEEMMTACQGVGGPGHKAR | P05888 | 2 |
| pep22049 | gag | C-terminus of p24[a] | 309-364 | 1260-1428 | ASQEVKNWMTETLLVQNANPDCKTILKALGPAATLEEMMTACQGVGGPGHKARVLA | P03366 | 2 |
| pep24153 | gag | C-terminus of p24[a] | 309-364 | 1260-1428 | ASQEVKNWMTETLLVQNANPDCKTILKALGPAATLEEMMTACQGVGGPGHKARVLA | P05960 | 2 |
| pep22050 | gag | C-terminus of p24[a] | 337-392 | 1344-1512 | LGPAATLEEMMTACQGVGGPGHKARVLAEAMSQVTNTATIMMQRGNFRNQRKMVKC | P03366 | 2 |
| pep22087 | pol | C-terminus of integrase[b] | 1373-1428 | 4415-4583 | QKQITKIQNFRVYYRDSRNPLWKGPAKLLWKGEGAVVIQDNSDIKVVPRRKAKIIR | P03366 | 3 |
| pep23273 | pol | C-terminus of integrase[b] | 1373-1428 | 4436-4604 | QNFRVYYRDNRDPIWKGPAKLLWKGEGAVVIQDNSDIKVVPRRKAKIIRDYGKQMA | P04588 | 3 |
| pep64646 | pol | C-terminus of integrase[b] | 1373-1428 | 4439-4607 | NFRVYYRDSRDPIWKGPAKLLWKGEGAVVIQDNSDIKVVPRRKAKIIRDYGKQMAG | Q75002 | 3 |
| pep65581 | pol | C-terminus of integrase[b] | 1373-1428 | 4448-4616 | VYYRDSRDPIWKGPAQLLWKGEGAVVIQDKGDIKVVPRRKAKIIRDYGKQMAGTDS | Q79666 | 3 |
| pep31722 | vpu | N-terminus of the cytoplasmic domain of vpu | 1-56 | 5674-5842 | MQSLEILAIVALVVAAILAIVVWTIVGIEIRKTLRQKKIDRLIDRIRERAEDSGNE | P12518 | 4 |
| pep24119 | vpu | N-terminus of the cytoplasmic domain of vpu | 29-81 | 5695-5854 | YRKILRQRKIDRLIDRLIERAEDSGNESEGEISALVEMGVEMGHHAPWDVDDL | P05919 | 4 |
| pep19035 | env | gp120, spanning V3 and the CD4 binding loop | 253-308 | 6548-6716 | LLNGSLAEEQIIIRTKNISDNTKNIIVQLKTPVNITCTRPNNNTRTSIHLGPGRAF | O70902 | 5 |
| pep77294 | env | gp120, spanning V3 and the CD4 binding loop | 281-336 | 6608-6776 | NTKTIIVQFKNPVKINCTRPNNNTRRSIHIGPGRAFYATGEIIGDTRKAHCNISEK | Q9QBZ0 | 5 |
| pep41884 | env | gp120, spanning V3 and the CD4 binding loop | 281-336 | 6611-6779 | AKTIIVHLNESVEINCTRPNNNVRRRHIHIGPGRAFYTGEIRGNIRQAHCNISRAK | P31872 | 5 |
| pep19036 | env | gp120, spanning V3 and the CD4 binding loop | 281-336 | 6632-6800 | LKTPVNITCTRPNNNTRTSIHLGPGRAFYATGDIIGDIRQAHCNISRTDWNKTLHQ | O70902 | 5 |
| pep23205 | env | gp120, spanning V3 and the CD4 binding loop | 309-364 | 6695-6863 | RRGIHFGPGQALYTTGIVGDIRRAYCTINETEWDKTLQQVAVKLGSLLNKTKIIFN | P04583 | 5 |
| pep22130 | env | gp120, spanning V3 and the CD4 binding loop | 309-364 | 6704-6872 | SIYIGPGRAFHTTGRIIGDIRKAHCNISRAQWNNTLEQIVKKLREQFGNNKTIVFN | P03378 | 5 |
| pep77300 | env | gp120 C-terminus, spanning V5 and the fusion peptide | 449-504 | 7130-7298 | LTIDGGEGNESETLRPGGGDMRDNWRSELYKYKVVKIEPLGVAPTKAKRQVVQREK | Q9QBZ0 | 6 |

## Supplementary Materials

## Table S1 (continued)

| | | | | | | | | |
|---|---|---|---|---|---|---|---|---|
| pep77300 | env | gp120 C-terminus, spanning V5 and the fusion peptide | 449-504 | 7130-7298 | LTIDGGEGNESETLRPGGGDMRDNWRSELYKYKVVKIEPLGVAPTKAKRQVVQREK | Q9QBZ0 | 6 |
| pep35894 | env | gp120 C-terminus, spanning V5 and the fusion peptide | 449-504 | 7133-7301 | TRDGGGDKNSTTEIFRPAGGNMKDNWRSELYKYKVVKIEPLGVAPTKAKRRVVQRE | P19549 | 6 |
| pep36409 | env | gp120 C-terminus, spanning V5 and the fusion peptide | 449-504 | 7139-7307 | DGGKNESEIEIFRPGGGDMRDNWRSELYKYKVVKIEPLGVAPTKAKRRVVQREKRA | P20871 | 6 |
| pep35924 | env | gp120 C-terminus, spanning V5 and the fusion peptide | 449-504 | 7142-7310 | GKEISNTTEIFRPGGGDMRDNWRSELYKYKVVKIEPLGVAPTKAKRRVVQREKRAV | P19550 | 6 |
| pep19042 | env | gp120 C-terminus, spanning V5 and the fusion peptide | 449-504 | 7148-7316 | NASAENYTFRPGGGDMRDNWRSELYKYKVVKIEPLGIAPTKTRRRVVEREKRAVGM | O70902 | 6 |
| pep64680 | env | gp120 C-terminus, spanning V5 and the fusion peptide | 449-504 | 7154-7322 | EPHSTKEIFRPEGGDMRDNWRSELYKYKVVEIKPLGVAPTKPKRRVVEREKRAALG | Q75008 | 6 |
| pep42484 | env | gp120 C-terminus, spanning V5 and the fusion peptide | 449-504 | 7157-7325 | TNGTEIFRPGGGDMRDNWRSELYKYKVVKIEPLGVAPTKAKRRVVQREKRAVGLGA | P35961 | 6 |
| pep31644 | env | gp120 C-terminus, spanning V5 and the fusion peptide | 477-532 | 7163-7331 | TETFRPGGGNMRDNWRSELYKYKVVKIELLGVAPTKAKRRVVQREKRAVGIGAVFL | P12489 | 6 |
| pep23962 | env | gp120 C-terminus, spanning V5 and the fusion peptide | 477-532 | 7166-7334 | EIFRPGGGDMRDNWRSELYKYKVVKIEPLGVAPTKAKRRVVQREKRAVGMLGAMFL | P05879 | 6 |
| pep77354 | env | gp120 C-terminus, spanning V5 and the fusion peptide | 449-504 | 7166-7334 | IDGKEILRPIGGDMRDNWRSELYKYKVVRIEPVGVAPTKAKRRVVQRAKRAVGMGA | Q9QBZ4 | 6 |
| pep41861 | env | gp120 C-terminus, spanning V5 and the fusion peptide | 477-532 | 7181-7349 | GGGDMRDNWRSELYKYKVVKIEPLGVAPTRAKRRVVQREKRAVGIGAVFLGFLGAA | P31819 | 6 |
| pep22106 | env | gp120 C-terminus, spanning V5 and the fusion peptide | 477-532 | 7184-7352 | GGDMRDNWRSELYKYKVVKIEPLGVAPTKAKRRVVQREKRAVGIGALFLGFLGAAG | P03377 | 6 |
| pep78238 | env | gp120 C-terminus, spanning V5 and the fusion peptide | 449-504 | 7187-7355 | GNMKDNWRSELYKYKVVEIEPLGVAPTKAKRQVVQREKRAAGLGALFLGFLGDSRE | Q9QSQ7 | 6 |
| pep23932 | env | gp120 C-terminus, spanning V5 and the fusion peptide | 477-532 | 7193-7361 | MRDNWRSELYKYKVVTIEPLGVAPTKAKRRVVQREKRAAIGALFLGFLGAAGSTMG | P05877 | 6 |
| pep41891 | env | gp120 C-terminus, spanning V5 and the fusion peptide | 477-532 | 7202-7370 | NWRSELYKYKVVKIEPLGVAPTKAKRRVVQREKRAVGAIGAMFLGFLGAAGSTMGA | P31872 | 6 |
| pep31614 | env | gp120 C-terminus, spanning V5 and the fusion peptide | 477-532 | 7211-7379 | SELYKYKVVKIEPLGVAPTKAKRRVVQREKRAVGLGALFLGFLGAAGSTMGAASLT | P12488 | 6 |
| pep35895 | env | gp120 C-terminus, spanning V5 and the fusion peptide | 477-532 | 7214-7382 | ELYKYKVVKIEPLGVAPTKAKRRVVQREKRAVGVIGAMFLGFLGAAGSTMGAASIT | P19549 | 6 |
| pep35925 | env | gp120 C-terminus, spanning V5 and the fusion peptide | 477-532 | 7226-7394 | YKVVKIEPLGVAPTKAKRRVVQREKRAVTLGAMFLGFLGAAGSTMGARSLTLTVQA | P19550 | 6 |
| pep42485 | env | gp120 C-terminus, spanning V5 and the fusion peptide | 477-532 | 7238-7406 | KIEPLGVAPTKAKRRVVQREKRAVGLGALFLGFLGAAGSTMGAASITLTVQARQLL | P35961 | 6 |
| pep23963 | env | gp120 C-terminus, spanning V5 and the fusion peptide | 505-560 | 7250-7418 | LGVAPTKAKRRVVQREKRAVGMLGAMFLGFLGAAGSTMGATSMALTVQARQLLSGI | P05879 | 6 |
| pep31645 | env | gp120 C-terminus, spanning V5 and the fusion peptide | 505-560 | 7250-7418 | LLGVAPTKAKRRVVQREKRAVGIGAVFLGFLGAAGSTMGASMTLTVQARLLLSGIV | P12489 | 6 |
| pep23122 | env | gp120 C-terminus, spanning V5 and the fusion peptide | 505-560 | 7259-7427 | APTRAKRRVVQREKRAVGTIGAMFLGFLGAAGSTMGAGSITLTVQARHLLSGIVQQ | P04579 | 6 |
| pep22107 | env | gp120 C-terminus, spanning V5 and the fusion peptide | 505-560 | 7268-7436 | KAKRRVVQREKRAVGIGALFLGFLGAAGSTMGARSMTLTVQARQLLSGIVQQQNNL | P03377 | 6 |
| pep31649 | env | gp41 coiled coil domain [c] | 617-672 | 7586-7754 | TAVPWNASWSNKSLEEIWDNMTWMEWEREIDNYTSLIYTLIEESQNQQEKNEQELL | P12489 | 7 |
| pep41866 | env | gp41 coiled coil domain [c] | 617-672 | 7601-7769 | NTSWSNKSFNEIWDNMTWMEWEREINNYTNLIYNLIEESQNQQEKNEQDLLALDKW | P31819 | 7 |
| pep22111 | env | gp41 coiled coil domain [c] | 617-672 | 7604-7772 | ASWSNKSLEQIWNNMTWMEWDREINNYTSLIHSLIEESQNQQEKNEQELLELDKWA | P03377 | 7 |
| pep35900 | env | gp41 coiled coil domain [c] | 617-672 | 7631-7799 | KIWNNMTWMEWEREIDNYTSLIYTLLEESQNQQEKNEQELLELDKWASLWNWFSIT | P19549 | 7 |
| pep19038 | env | gp120, spanning V3 and the CD4 binding loop | 337-392 | 6833-7001 | VVTQLGIHLNNRTISFKPNSGGDMEVRTHSFNCRGEFFYCNTSGLFNSSWEMHTNY | O70902 | None |

**Supplementary Materials**

**Table S2. Peptides that had a significant association between viral load set point and median antibody reactivity (RV217 Cohort).** The table shows the amino acid sequences of motif 1 and the nine peptides that had a significant association between viral load set point and median antibody reactivity. All nine peptides were located in the N-terminus of the HIV gag protein (p17, amino acids 29-84). Amino acids that differ from peptide 20344 are shown in red font.

| | | estimate | std error | statistic | p-value |
|---|---|---|---|---|---|
| Motif for cluster 1 | 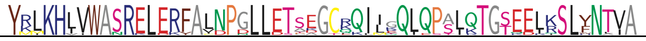 | | | | |
| pep_ 20344 | YRLKHLVWASRELERFALNPGLLETPEGCLQIIEQIQPAIKTGTEELKSLFNLVAV | -0.6764 | 0.16076 | -4.20776 | 0.00010 |
| pep_ 64581 | YMLKHLVWANRELEKFALNPDLLDTSAGCKQIIKQLQPALQTGTEELKSLFNTVAT | -0.94480 | 0.23585 | -4.00586 | 0.00020 |
| pep_ 31689 | YRLKHLVWASRELERFALNPGLLETSDGCKQIIGQLQPAIRTGSEELRSLFNTVAT | -0.6718 | 0.17599 | -3.81763 | 0.00037 |
| pep_ 64598 | YMLKHLVWANRELEKFALNPDLLDTSAGCKQIIKQLQPALQTGTEELKSLFNTVAT | -0.81228 | 0.21462 | -3.78462 | 0.00041 |
| pep_ 77406 | YRLKHLVWASRELERFALNPNLLETVEGCRQIIRQLQPSLQTGSEELRSLFNTVAT | -0.65455 | 0.17871 | -3.6624 | 0.00059 |
| pep_ 23294 | YRLKHIVWASRELERYALNPGLLETSEGCKQIIGQLQPAIQTGTEELRSLYNTVAT | -0.7135 | 0.19603 | -3.64006 | 0.00064 |
| pep_ 76931 | YRLKHLVWASRELERFALNPDLLETADGCQQILGQLQPALKTGTEDLQSLYNTIAV | -0.6463 | 0.18486 | -3.49614 | 0.00099 |
| pep_ 19339 | YRLKHLVWASRELERFALDPGLLETSEGCRKIIGQLQPSLQTGSEELKSLYNTIAV | -0.64451 | 0.18508 | -3.4823 | 0.00103 |
| pep_ 17419 | YMMKHLVWASRELERFALDPGLLETSEGCKQIMKQLQPALQTGTKELISLHNTVAT | -0.60477 | 0.18329 | -3.29938 | 0.00178 |

**Supplementary Materials**

**Table S3. Characteristics of vpu peptides that had higher reactivity in HIV controllers compared to non-controllers who were virally suppressed on antiretroviral therapy.** The table shows the amino acid sequences of the two vpu peptides that had significantly higher antibody reactivity in HIV controllers compared to non-controllers who were virally suppressed on antiretroviral therapy (Discovery Cohort). The sequences of these two peptides are compared to the amino acid sequences of groups of 2-3 overlapping peptides that were more frequently targeted in an antibody-dependent cellular cytotoxicity (ADCC) assay by long-term slow progressors (56), and the amino acid sequence of a peptide targeted in ADCC assays by elite controllers (57). The sequence for the vpu19 peptide used in the previous study is reported by Stratov et al. The amino acid motif for cluster 4 in vpu identified in this report is also shown (bottom row). The portions of the two vpu peptides identified in this report that contain the amino acid motif are shown in blue font. The amino acids that differ in the overlapping region of these two peptides are underlined. Amino acid differences between the vpu peptides identified in this report and vpu peptides identified in the prior report are shown in red font.
Abbreviations: EC: elite controllers; VC: viremic controllers; LTSP: long-term slow progressors.

| Peptide ID | Source | Study group | Amino acid position | HXB2 coordinates | Amino acid sequence |
|---|---|---|---|---|---|
| pep_ 31722 | This report | EC/VC | vpu: 1-56 | 5674-5842 | MQSLEILAIVALVVAAILAIVVWTIVGIEIRKTLRQKKIDRLIDRIRERAEDSGNE |
| vpu 7,8 | Wren et al | LTSP | | | VVWTIVFIEYRKILRQRKI |
| vpu 10, 11, 12 | Wren et al | LTSP | | | ILRQRKIDRLIDRIRERAEDSGN |
| pep_ 24119 | This report | EC/VC | vpu: 29-81 | 5695-5854 | YRKILRQRKIDRLIDRLIERAEDSGNESEGEISALVEMGVEMGHHAPWDVDDL |
| vpu 18, 19 | Wren et al | LTSP | | | SALVEM----GHHAPWDVDDL |
| vpu 19 | Madhavi et al | EC | | | EM----GHHAPWDVDDL |
| Motif for cluster 4 | This report | | | | 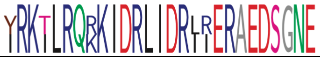 |

## References

Statov, I., et al., Robust NK cell-mediated human immunodeficiency virus (HIV)-specific antibody-dependent responses in HIV-infected subjects. J Virol, 2008. 82(11):5450-5459.
